# Knowledge, Attitude, and Practices of Dentists in Caribbean Countries during the COVID-19 Pandemic: A Multicenter Cross-Sectional Study

**DOI:** 10.3390/dj9110133

**Published:** 2021-11-15

**Authors:** Ramaa Balkaran, Meghashyam Bhat, Shivaughn Marchan, William Smith

**Affiliations:** 1 School of Dentistry, Faculty of Medical Sciences, The University of the West Indies St. Augustine, St. Augustine, Trinidad and Tobago; shivaughn.marchan@sta.uwi.edu (S.M.); william.smith@sta.uwi.edu (W.S.); 2 Australian Research Centre for Population Oral Health (ARCPOH), School of Dentistry, University of Adelaide, Adelaide, SA 5005, Australia; meghashyam.bhat@adelaide.edu.au

**Keywords:** COVID-19, Caribbean, dentists, knowledge, practice

## Abstract

Background: The COVID-19 pandemic has affected dentists globally, both financially and mentally. This study aimed to determine the knowledge, attitude, and practices of dentists in Caribbean countries during the COVID-19 pandemic. Methods: A non-probability sample was obtained from dentists in more than ten different Caribbean countries. They were invited to complete a self-reported questionnaire, which was conducted from December 2020 to March 2021. Ethics approval was sought and an exemption was received from the UWI ethics committee. Results: One hundred and fifty-two dentists responded. More than one-third (38.8%) were in the >35–45 age group, and 58.6% were females. Most (84.9%) were general dentists and 75% were stressed by the COVID-19 situation with 80.9% being affected financially. The majority, 94.7%, believed that the highest risk of transmission of COVID-19 was via aerosol-generating procedures and 87.5% were worried about contracting it clinically. The majority (69.1%) were willing to receive the vaccine, the main reason reported for vaccine hesitancy was due to the possible side effects (35.3%). Most (75%) consumed alcohol. When the locus of control was determined, 54.6% felt they were in control of protecting themselves while 52% felt that external factors controlled their lives. Conclusions: The findings suggest that most dentists in the Caribbean were knowledgeable about COVID-19 and followed current guidelines in their practice and were willing to receive the vaccine.

## 1. Introduction

The pandemic caused by the novel coronavirus disease of 2019, commonly known as COVID-19, changed all aspects of daily lives, including that of the practice of dentistry. Dentists adapted their practices based on emerging data, which involved triaging and incorporated the use of new forms of personal protective equipment (PPE). This highly contagious virus was caused by a recent strain of the novel coronavirus termed SARS-CoV-2, which began in December 2019 and soon became a pandemic by March 2020 [1]. The virus causes a range of respiratory symptoms and there have been several new variants of concern (VOC), which are mainly spread through droplet infection [1]. Currently, there are four VOCs, which have been labelled by WHO as Alpha, Beta, Gamma, and Delta, and existing recommended strategies have been shown to work against them [1].

Generally, these strategies consist of public health initiatives to reduce the burden on health systems, and each government employed various social measures, such as social distancing, hand hygiene, physical isolation, and wearing of face masks. Vaccines were approved for emergency use worldwide by November 2020 and became available to Caribbean countries by February 2021. Although COVID-19 vaccinations showed a reduction in the transmission of SARS-CoV-2 and emerging variants, they worked best when social distancing measures were also applied [2]. 

Dentists and their teams who are constantly exposed to the oral cavity are considered to be at a high risk of exposure to COVID-19 via aerosols and droplets. Furthermore, there has been confirmation of transmission via persons who have been asymptomatic or pre-symptomatic [3]. Moreover, dentists have reported oral signs of COVID-19 disease both before and following diagnosis, in which this virus can cause cutaneous and mucosal lesions as secondary manifestations such as aphthous-like lesions or erosions [4]. Other reported oral symptoms have included xerostomia, halitosis, parotitis, and sialadenitis [5]. Critically ill patients in the intensive care units also showed oral complications such as perioral pressure ulcers, oral candidiasis, herpetic and hemorrhagic ulcers, and acute onset macroglossia [6]. This underscores the necessity for stringent infection control and adherence to guidelines, as information on COVID-19 has been dynamic since its emergence.

Worldwide, certain guidelines specifically targeted the practice of dentistry such as screening, following universal precautions, triaging, and management with minimally invasive procedures [7]. Although there were no universal guidelines, only emergency treatment was deemed necessary from March 2020 and all elective procedures were delayed until June 2020 [8,9,10]. The Caribbean was no exception, and although every island has its own dental regulatory body, their published guidelines were based on the country’s restrictions by their governments to dentists, and were largely consistent with international bodies. Here, the Caribbean may be geographically defined as the islands between “the Florida Peninsula and the coast of Venezuela, the Greater and Lesser Antilles on the Caribbean Sea, and secondly, the mainland” [11]. Politically, these islands are individually governed with some having American and European ties. Additionally, the English-speaking countries have formed a Caribbean Community (CARICOM) that supports each other economically and allows trade among them [11].

These guidelines for dentistry involved types of patient care permitted, such as emergency/urgent treatment, social distancing practices, sanitization procedures, and the use of PPE. The latter has also led to increased cost of treatment This impacted patients who were facing pandemic-related uncertainty, and coupled with the increased dental costs, many of them delayed their dental care, which also economically challenged dentists [12].

Given the fact that the pandemic has affected dentists financially and mentally, it is important to understand coping mechanisms during this stressful period. Consequently, the locus of control of the participants was assessed using a global scale that has been previously validated [13]. This concept defines whether or not a person believes that they are in control of outcomes in various circumstances in their life, or if they are a consequence of an external influence. 

This study aimed to determine the knowledge, attitude, and practices of dentists in Caribbean countries during the COVID-19 pandemic.

## 2. Materials and Methods

A non-probability sample was obtained from dentists in more than ten different Caribbean countries via an online platform for three months from December 2020 to March 2021. The questions were based on a previously piloted questionnaire that had been subjected to face validity [14]. The questions were chosen based on common issues that faced dentists in the Caribbean, such as, stress, work–life balance, and how related factors affected them. Any ambiguous questions were eliminated or re-worded during the pilot survey. A link to the online survey tool was sent to all dentists in the Caribbean via email, through their country’s respective dental associations and dental councils. The email invited the participants to respond to the anonymous questionnaire. Participants were given a time frame to respond and were reminded to respond at monthly intervals. The Checklist for Reporting Results of Internet E-Surveys (CHERRIES) was used from the development to the analysis of the web-based survey to ensure that participants did not complete the survey multiple times, and this was carried out through an analysis of the IP addresses [15].

The questionnaire (Appendix A) comprised a total of 44 questions, 3 of which belonged to the cognitive domain, 5 to the affective domain, and 4 to the psychomotor domain. Further included were questions on general demographics, dental training, internal and external locus of control, coping patterns, and current infection control practices in the dental setting. A 5-point Likert scale from 1 (strongly disagree) to 5 (strongly agree) was used for questions related to the pandemic and locus of control. Additionally, a 3-point Likert scale from 1 (not worried at all) to 3 (worried all the time) was used for various concerns in daily life. The Likert scale was chosen since it is a universal method of collecting data and is aligned with a vast array of scientifically vetted articles. It is used in long multi-item questionnaires and prevents respondents from being overwhelmed and has a neutral option that prevents skewing of results.

Data were organized and entered using Microsoft Excel (Microsoft Corp., Redwood, WA, USA) and analyzed using SPSS (software package version 22 IBM, Armonk, NY, USA). Descriptive analyses, cross-tabulations, and Pearson’s correlation at the 0.05% level of significance were assessed for the multiple variables to determine the association between the variables. An ethics exemption was obtained from the UWI ethics committee and approval was granted (CREC-SA.0665/01/2021), as the research did not involve any invasive procedures and was carried out as per the Helsinki declaration.

## 3. Results

The population of respondents was comprised of 152 persons. More than one-third (38.8%) were in the greater than 35–45 age group, and 58.6% were females. Most were general dentists (84.9%) (Table 1).

**Figure 1:**
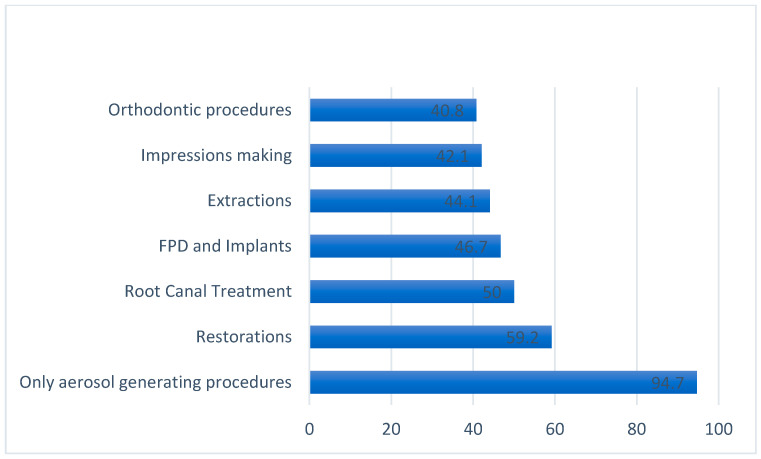
Participants’ knowledge of procedures that carry the highest risk of transmission of COVID-19.

Respondents were from eight different Caribbean countries, and of these, most (75%) stated that they were stressed by the COVID-19 situation, with 80.9% being affected financially by the pandemic. The vast majority, 94.7%, believed that the highest risk of transmission of COVID-19 was via aerosol-generating procedures (Figure 1). 

Moreover, 87.5% were worried about contracting it in a clinical setting (Table 2).

The majority (69.1%) were willing to receive the vaccine, and the main reason the remaining dentists reported hesitance to receive the vaccine was due to adverse effects (35.3%). Most (75%) consumed alcohol and 14.5% stated that it did not help them cope with stress. The majority (78.9%) called friends and colleagues over phone/video call as a coping mechanism (Table 3). 

When the locus of control was determined, just over half (54.6%) felt they were in control of protecting themselves while 52% felt that external factors controlled their lives. Most (73.7%) followed triaging, and since COVID-19, the majority (94.1%) used personal protective equipment (Table 4) 

**Figure 2:**
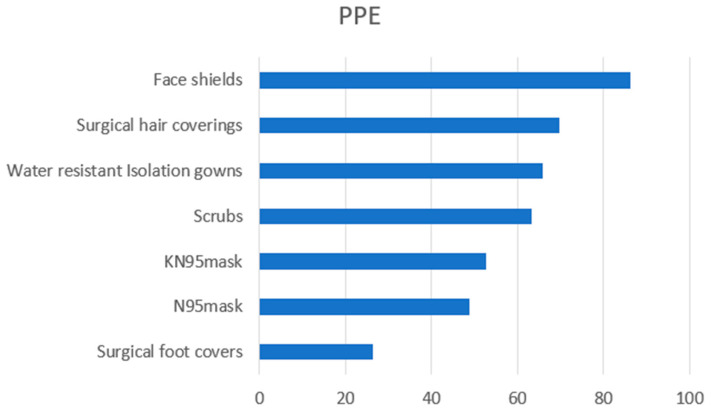
Personal Protective Equipment (PPE) used since COVID-19.

Additionally, face shields were the largest (86.2%) reported use of PPE (Figure 2).

## 4. Discussion

Dentists were considered to be at high risk of exposure to this virus, amongst all healthcare workers, from the onset of the pandemic spread of COVID-19 [15,16]. Worldwide, various governments advised dentists on what treatment should be performed in the interest of the safety of public health [15]. At the start of the pandemic, many regulatory bodies within the Caribbean suggested or mandated urgent or emergency care [17]. In the Caribbean, dentists have been performing emergency treatment only, such as extractions, and then some countries changed this policy as the infection rate decreased; while others resumed routine treatment, others have not been able to do this given the current crisis in their countries [8,9,10,17].

Whilst this decision was made to aid in the reduction of the spread of the virus, even short-term interruptions in the dental practice led to economic concerns for dentists and their staff [18]. In our study, the majority were affected financially and were worried about meeting their financial commitments. In addition to the financial concerns, closure of dental practices can also have adverse patient outcomes. Research has shown that the mortality rate is higher in those with periodontal disease, which underscores the need for improved oral hygiene to reduce the bacterial load and respiratory complications, especially in the elderly and warded patients [19].

Additionally, the transmission of COVID-19 via droplets and aerosols from infected patients in dental settings has ensured that dentists adhere to strict infection control measures to ensure the prevention of its spread [20]. This has led to the recommendation for increased use of costly and extra PPE such as N95 respirators [21].

It is noteworthy that in this study, the level of concern for the variables of “getting infected by allied healthcare personnel at the private practice” and “getting infected from patients attending the dental clinical setting” was found to be statistically significant for persons who were stressed. Although there is an increased cost associated with the use of enhanced PPE by dentists, research has shown that the natural infection rates with SARS-CoV-2, among dentists, were significantly higher compared to the general population before its use [22]. In a recent study, dental students were afraid of transmitting the virus to their families due to the perception that the standard precautions used in dentistry were unsafe when used with COVID-19 patients [23,24]. Conversely, a recent study concluded that standard infection control practices were sufficient to protect dental personnel and patients from exposure to potential pathogens and dental treatment was not a factor in increasing the risk for transmission of SARS-CoV-2 in asymptomatic patients [25]. Recent research has also suggested that although natural infection provided some immunity to the virus, it emphasized the importance of vaccinations in achieving herd immunity [22]. Our study was conducted between December 2020 to March 2021, and vaccines were only available in certain Caribbean countries since February 2021. Furthermore, vaccines have been shown by the scientific community to be the most effective of the existing solutions in the control of the COVID-19 pandemic [26]. The vaccines that were used were approved by different countries based on their respective governments’ research and procurement ability [27]. Vaccine hesitation has been reported worldwide, even amongst healthcare professionals, and this is largely due to the novelty of these vaccines and reported side effects along with the spread of misinformation. The vast majority of the respondents claimed that they used social media more since the pandemic; however, only one-quarter was unsure about taking the vaccine. This is a positive sign given that vaccine hesitancy is a public health issue worldwide, and recent research found a significant relationship between social media and public doubts of vaccine safety, especially concerning disinformation on vaccines [28].

At the time that this study was conducted, there were only three VOCs; however, with the emergence of other variants of interest, such as Lambda and Mu [1], dentists may be concerned whether new guidelines and limited movements will be reinstituted. This may lead to further fear and anxiety since fear of the unknown is a major course of stress for healthcare workers [29]. 

Dentists have also been shown to experience mental health issues in some form of symptoms and stress due to the effects of the pandemic in their daily work routine [30,31]. This research assessed the internal and external locus of control of dentists who stated that they were stressed by the COVID-19 situation. Just over half (52%) of the participants felt that external factors controlled their life, while a similar percentage (54.6%) agreed that they were in control of protecting themselves. This study also found that the majority of participants were stressed about the COVID-19 situation, which was similar to the findings by León-Manco et al. 2021 [32] and Bastani et al. 2021 [33]. Although they were stressed, most participants of this study had social support systems and mechanisms to deal with the stress, which mainly involved calling friends and colleagues over phone/video calls and engaging in hobbies.

Additionally, the COVID 19 pandemic brought about innovation in terms of utilizing virtual strategies such as teledentistry and online counselling, which are effective measures of aiding in service delivery and to help one cope during this pandemic with reduced physical interaction [33]. Dentists must have coping mechanisms to manage their stress during this ongoing pandemic. In our study, the most common coping mechanisms participants stated were calling friends and colleagues over phone/video calls or using social media. The latter was similar to other research where electronic media and entertainment were reported to be common stress busters [34].

### Limitations

The information on COVID-19 is constantly evolving, which may alter the attitude of the participants if conducted at a later time. Further, at the time that the study was undertaken, it was during a period when not much was known regarding the pandemic.

## 5. Conclusions

The findings suggest that dentists are knowledgeable about the spread of COVID and are following international guidelines in their infection control Although most dentists were stressed by the COVID-19 situation, they had social support systems and coping mechanisms. They are also willing to receive the vaccine. 

## Figures and Tables

**Table 1:** Demographics of participants.

Variables	n (152)	Valid%
Gender		
Male	63	41.4
Female	89	58.6
Age		
25–35	42	27.6
35–45	59	38.8
>45–55	39	25.7
>55–65	11	7.2
>65	1	0.7
Home living arrangements		
Live alone	23	15.1
Live with elderly parents	35	23.0
Live with partner/spouse and children	83	54.6
Other	11	7.2
Specialty		
None—General Dentist	129	84.9
Restorative	4	2.6
Oral and maxillofacial surgery	4	2.6
Pediatric dentistry	3	2.0
Dental public health	3	2.0
Orthodontics and dentofacial orthopedics	3	2.0
Periodontics	3	2.0
Maxillofacial pathology	1	0.7
Other	1	0.7
Endodontics	1	0.7
Qualification		
DDS	119	78.3
MSc	31	20.4
PhD	2	1.3
Length of time in dental practice		
<5 years	49	32.2
5–10 years	16	10.5
10–20 years	55	36.2
>20 years	32	21.1
Alcohol consumption		
Yes	116	76.3
No	36	23.7
Cigarette Smokers		
Daily	6	3.9
Weekly	20	13.2
Monthly	6	3.9
N/A	120	78.9
Income		
1500–2K US	17	11.2
2001–4k	24	15.8
4001–6k	15	9.9
>40k/>6k	24	15.8
Prefer not to say	72	47.4
Work country		
Trinidad and Tobago	118	77.6
Jamaica	10	6.6
Barbados	14	9.2
Bahamas	2	1.3
Puerto Rico	3	2.0
Dominican Republic	2	1.3
Montserrat	2	1.3

**Table 2:** Levels of concern among participants who were stressed.

How Worried Are You about?	Not Worried at All (%)	Sometimes Worried (%)	Worried All the Time (%)	Pearson’s R Significance Values
Getting infected by allied healthcare personnel at the private practice	9.6	62.3	28.1	0.000
Infecting my family	5.3	34.2	60.5	0.000
Patient-to-patient transmission	32.5	43.9	23.7	0.196
Being laid off from work	32.5	21.9	10.5	0.222
Meeting monthly financial commitments	14.9	41.2	40.4	0.08
Pandemic has affected your practice financially	87.7	7.9	4.4	0.001
Your country should it escalate	1.8	46.5	51.8	0.000
Infected from patients attending your dental clinical setting	7.9	56.1	36.0	0.001

**Table 3:** Ways of coping.

Coping Mechanism	n *	Valid%
Calling Friends and colleagues over phone/video call.	120	78.9
Engage in hobbies.	94	61.8
Continue to keep abreast with academic activities online.	65	42.8
Meeting people by following social distancing norms	46	30.3
Use social media more after pandemic	107	70.4

* Participants were allowed to state multiple responses.

**Table 4:** Strategies participants practiced since COVID-19.

Parameters	n *	Valid%
Use personal protective equipment such as dental goggles, masks, and gloves	143	94.1
Put facemask on known or suspected patient	57	37.5
Avoid moving and transporting patients out of their area unless necessary	86	56.6
All health staff members wear protective clothing	140	92.1
Place known or suspected patients in adequately ventilated single rooms	35	23.0

* Participants were allowed to state multiple responses.

## Data Availability

The data presented in this study are available on request from the corresponding author. The data are not publicly available due to privacy considerations.

## Appendix A

The Knowledge: attitude and practices of dentists in CARICOM countries during the COVID-19 situation.

This questionnaire is intended for dentists working in the Caribbean Islands. Completion of this survey is recommended as it will provide information on the knowledge, attitude and practices of dentists during the COVID-19 pandemic and shall also help in recommending health policies for dentists. Your privacy is important. Participants’ identity will not be identified in anyway. The information to be collected during this study will be confidential and will be strictly used for research purposes only. If you have questions about your rights as a person who is taking part in a research study, you may contact the Campus Research Ethics Committee by email at Campusethics@sta.uwi.edu The study involves completing the attached questionnaire. Your participation will take approximately 10–15 minutes. By continuing to the next page, you indicate your consent and willingness to take part in the survey.

Q1. Please state if you are stressed with the COVID-19 situation in the Caribbean islands?
  - yes
  - no

Q2. Are you worried about getting infected from COVID-19 at work?
  - yes
  - no

Q3. How worried are you about getting infected from COVID-19 by an allied health care (e.g., DSA) personnel working in private clinic? Please rate on a scale from 1–3:
  - Not worried at all
  - Sometimes worried
  - Worried all the time

Q4. How worried are you about carrying the infection and infecting your family? Please rate on a scale from 1–3
  - Not worried at all
  - Sometimes worried
  - Worried all the time

Q5. How worried are you about patient-to-patient transmission in the dental clinic? Please rate on a scale from 1–3:
  - Not worried at all
  - Sometimes worried
  - Worried all the time

Q6. Are you worried about being laid off from work? Please rate on a scale from 1–3:
  1. Not worried at all
  2. Sometimes worried
  3. Worried all the time
  4. N/A

Q7. Are you worried about what will happen to your country and if they would be in a position to cope with the crisis should it escalate? Please rate on a scale from 1–3:
  - Not worried at all
  - Sometimes worried
  - Worried all the time

Q8. Are you worried about getting infected from patients attending your private practice? Please rate on a scale from 1–3:
  - Not worried at all
  - Sometimes worried
  - Worried all the time

Q9. Which procedure do you feel carries the highest risk of transmission of COVID-19?
  1. Restorations
  2. Root Canal Treatment
  3. Extractions
  4. Impressions making
  5. FPD and Implants
  6. Orthodontic procedures
  7. Only aerosol generating procedures
  8. All of the above

Q10. Are you following the telephone triaging approach when dealing with patients at your private practice?
  - yes
  - no

Q11. Do you agree that the COVID-19 changed your outlook towards life? Please rate on a scale from 1–5:
  - strongly disagree
  - disagree
  - neutral
  - agree
  - strongly agree

Q12. Do you feel as though you don’t have control in protecting yourself from the COVID-19 situation? Please rate on a scale from 1–5:
  - strongly disagree
  - disagree
  - neutral
  - agree
  - strongly agree

Q13. Do you believe that you are more mindful and appreciate what you have due to the uncertainty in life? Please rate on a scale from 1–5:
  - strongly disagree
  - disagree
  - neutral
  - agree
  - strongly agree

Q14. Do you believe that external factors control your life? Please rate on a scale from 1–5:
  - strongly disagree
  - disagree
  - neutral
  - agree
  - strongly agree

Q15. Do you feel that you are in control of protecting yourself from the COVID 19 situation? Please rate on a scale from 1–5:
  - strongly disagree
  - disagree
  - neutral
  - agree
  - strongly agree

Q16. Do you agree that after the COVID-19 situation, you wash/sanitize your hands more frequently? Please rate on a scale from 1–5
  - strongly disagree
  - disagree
  - neutral
  - agree
  - strongly agree

Q17. Do you feel social distancing can help in preventing the spread of infection
  - Yes,
  - No,
  - Don’t know

Q18. Do you practice social distancing in public and at work?
  - Yes
  - No
  - Unable to do so

Q19. Which of the following do you practice, since COVID-19?
  1. Personal protective equipment such as dental goggles, masks, and gloves
  2. Put facemask on known or suspected patient
  3. Avoid moving and transporting patients out of their area unless necessary
  4. All health staff members wear protective clothing
  5. Place known or suspected patients in adequately ventilated single rooms

Q20. Do you receive social support?
  - Yes
  - No

Q21. If yes from whom (tick all that apply)
  1. Friends and university colleagues
  2. Parents and relatives
  3. Spouse and children
  4. Government Helpline
  5. Social Media
  6. Not Applicable

Q22. Has your fitness routine been affected by the COVID-19 pandemic?
  - Yes
  - No
  - To some extent.

Q23. How do you cope socially?
  - Calling Friends/family and colleagues over phone/video call
  - Engage in hobbies
  - Continue to keep abreast with academic activities online.
  - Meeting people by following social distancing norms.

Q24. Do you use social media more after the COVID-19 pandemic began?
  - Yes
  - No
  - Same as before

Q25. Which form of social media so you use more often
  1. Digital social media (Facebook, Instagram, Twitter)
  2. Newspapers
  3. Television/radio
  4. same

Q26. Do you consume alcohol
  - Yes
  - No

Q27. Have you consumed alcohol in the past one year?
  - Yes
  - No
  - Not Applicable

Q28. If you consume how often do you consume and how much
  1. Every day (60 mL or more 1–2 beers)
  2. Alternate days (60 mL or more 1–2 beers)
  3. Twice a week (60 mL more 1–2 beers)
  4. Once a week
  5. Once in two weeks
  6. Once a month
  7. Occasionally
  8. Not Applicable

Q29. Do you feel your alcohol helps you cope with stress in the COVID-19 situation or otherwise?
  - Yes
  - No
  - Not Applicable

Q30. Has your consumption of alcohol increased after start of the COVID-19 pandemic?
  - Yes
  - No
  - Not Applicable

Q31. Do you smoke and have you smoked in the past one year?
  - Yes
  - No
  - Not Applicable

If yes: How often do you smoke?
  1. Daily
  2. Weekly
  3. Monthly
  4. Not Applicable

If you smoke daily: How many cigarettes do you smoke?
  1. 1–2 per day
  2. 3–5 per day
  3. More than 5 cigarettes per day.
  4. Not Applicable

Q32. Do you feel smoking helps you cope with stress?
  - Yes
  - No
  - Not Applicable

Q33. Has your smoking pattern increased after start of the COVID-19 pandemic?
  - Yes
  - No
  - Not Applicable

Q34. Do you use marijuana?
  - Yes
  - No

If Yes
  1. Regularly
  2. Occasionally
  3. Not Applicable

Q35. Do you feel the development of a COVID-19 vaccine can help in controlling the COVID-19 pandemic?
  - Yes
  - No
  - Don’t Know

Q36. Are you willing to receive the COVID-19 vaccine when introduced?
  - Yes
  - No
  - Unsure

Q37. If you received the vaccine, would you feel safer and stress free in practicing dentistry?
  - Yes
  - No
  - Unsure

Q38. What is your gender?
  - Male
  - Female

Q39. How old are you?
  1. 25–35
  2. >35–45
  3. >45–55
  4. >55–65
  5. >65 Multiple Choice

Q40. What are your home/living arrangements?
  1. Live alone
  2. Live with elderly parents
  3. Live with partner/spouse and children
  4. Other

Q41. Did you receive any training (courses/online training) on dealing with the COVID-19 situation?
  - Yes
  - No

Q42. What is your specialty (according to the American Dental Association classification)
  1. None-General Dentist
  2. Orthodontics
  3. Pediatric dentistry
  4. Periodontology
  5. Restorative
  6. Maxillofacial pathology
  7. Endo
  8. Radio
  9. Oral and maxillofacial surgery
  10. Dental Public Health
  11. Other

Q43. What is your highest academic degree?
  1. DDS (or equivalent)
  2. M.Sc. (or equivalent)
  3. PhD (or equivalent)

Q44. In which country do you work?
  1. Trinidad and Tobago
  2. Jamaica
  3. Barbados
  4. Bahamas
  5. St. Lucia
  6. Guyana
  7. Puerto Rico
  8. Dominican Republic
  9. Montserrat
  10. Other ---------------------------------

Q45. How many years have you been in private practice?
  1. <5
  2. 5–10
  3. 10–20
  4. >20

Q46. What is your monthly Income in TTD/USD?
  1. 15–2 k US
  2. 2–4 k US
  3. 4–6 k US
  4. Greater than 6
  5. Prefer not to say
